# The association between lymphocyte to high‐density lipoprotein ratio and depression: Data from NHANES 2015–2018

**DOI:** 10.1002/brb3.3467

**Published:** 2024-03-11

**Authors:** Junzhi Chen, Yan Huang, Xiaolin Li

**Affiliations:** ^1^ Division of Nephrology South China Hospital of Shenzhen University Shenzhen China; ^2^ National Clinical Research Center for Kidney Disease, State Key Laboratory of Organ Failure Research Nanfang Hospital Southern Medical University Guangzhou China

**Keywords:** depression, immune dysfunction, inflammation, lymphocyte to high‐density lipoprotein ratio

## Abstract

**Introduction:**

The relationship of lymphocyte to high‐density lipoprotein ratio (LHR) with depression remains uncertain. We aimed to evaluate the association between LHR and depression in US adults.

**Methods:**

In this cross‐sectional study, a total of 4216 participants were enrolled from the National Health and Nutrition Examination Survey (2015–2018). Depressive symptoms were measured with the Patient Health Questionnaire‐9 (PHQ‐9). Participants were classified as having depression if PHQ‐9 scores were ≥10. Multiple logistic regression models were used to explore the relationship between the LHR and depression.

**Results:**

Overall, the LHR was significantly associated with depression (per standard deviation increment; adjusted odds ratio (OR), 1.31; 95% confidence interval (CI) [1.14, 1.50]) after adjusted potential variables. Interactions between LHR with metabolic syndrome (MetS) and body mass index (BMI) on the risk of depression were found in stratified analysis (*p* for interaction < .05).

**Conclusions:**

A higher level of LHR was significantly associated with higher odds of having depression in US adults, and it was strengthened in participants with MetS or BMI ranging from 25 to 30 kg/m^2^.

## Graphical abstract

A higher level of lymphocyte to high‐density lipoprotein ratio (LHR) was significantly associated with higher odds of having depression. LHR may be a novel predictor of depression. LHR is an inexpensive marker for inflammation and can be easily acquired in clinical practice.

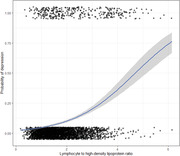

## 1 INTRODUCTION

Depression is a common psychiatric illness and is a major public problem worldwide. There were 350 million people of all ages suffer from depression globally according to the World Health Organization (WHO) (Al‐Khatib et al., 2022). In the United States, the prevalence of depression was about 7.5%, with nearly 17.3 million adults having experienced at least one major depression in 2017 (Shi et al., 2021; K. Wang et al., 2021). It is the leading cause of disability and is a major contributor to the overall global burden of disease (WHO, 2020). Previous studies have shown that depression is related to an increased risk of diabetes (Mezuk et al., 2008; Nouwen et al., 2010) cardiovascular disease (CVD) (Carney and Freedland, 2017; Musselman et al., 1998), Parkinson's disease (S. Wang et al., 2018), metabolic disease (Hidese et al., 2018), dementia (Byersand Yaffe, 2011), and cancer (Wakefield et al., 2015), all of which are in turn associated with increased risk of mortality (Fiske et al., 2009). In particular, the risk of early death in depressed patients was 4 times that of nondepressed patients (Wulsin et al., 1999), which are mainly caused by CVDs according to previous studies (Heiskanen et al., 2006; Osby et al., 2001). As such, it is important and urgent to develop an effective method to explore inexpensive and easily available biomarkers, resulting in an accurate diagnosis.

A growing body of studies suggests that inflammation and immune dysfunction are considered as crucial mechanisms in depression (Zhu et al., 2023), and the role of neuroimmune dysfunction in the inflammatory process is emphasized (Shan et al., 2022). Previous studies have reported that serum cytokines (Harsanyi et al., 2023), C‐reactive protein(CRP) (Horna et al., 2018), high‐density lipoprotein cholesterol (HDL‐C) (Akboga et al., 2016; Wu et al., 2013), complete blood count test indicators (Zhu et al., 2023) including lymphocytes, neutrophils, and platelets are associated with inflammation and oxidative stress. Among them, serum cytokines are expensive and not easily accessible in clinical practice. Lymphocyte counts are one of the important circulating leukocytes, mainly mediating the adaptive immune response and collaborating with innate immunity. HDL‐C is responsible for reverse cholesterol transport in the human body and is known for its several protective effects such as anti‐inflammatory, antioxidant, and antithrombotic (Akboga et al., 2016; Wu et al., 2013). Moreover, HDL‐C also plays an important role in regulating both adaptive and innate immune responses through the efflux of cholesterol from plasma cell membranes with the consequent disruption of lipid rafts and the interaction with the cholesterol transporters present in the plasma membrane (Fernandes das Neves et al., 2021), linking various pathways to inflammation and immune response (Bensinger et al., 2008). As for the complex interactions between lymphocytes and HDL‐C, the combined indicator lipoprotein ratio (LHR) might be more reliable in reflecting the inflammation level and the body's immunity than a single parameter.

Lymphocyte to high‐density LHR was proved to be a new marker of inflammation (H. Chen et al., 2019; T. Chen et al., 2020) and associated with metabolic syndrome (MetS, a prothrombotic and proinflammatory state) (H. Chen et al., 2019; T. Chen et al., 2020), cardiovascular risk factors (H. Chen et al., 2019), and sepsis (Liu et al., 2023). Moreover, LHR was superior to high‐sensitivity CRP (hsCRP) and lymphocyte counts in diagnosing the presence of MetS (H. Chen et al., 2019). A longitudinal study with 1194 participants in China has reported that LHR might be an effective predictor of newly diagnosed MetS and superior to platelet‐to‐lymphocyte ratio after a 4.66‐year follow‐up (Yu et al., 2021). To our knowledge, the association between LHR and depression is still unclear. Therefore, this study aims to explore the relationship between LHR and depression.

## 2 MATERIAL AND METHODS

### 2.1 Design and participants

This study was a cross‐section study. A total of 4216 populations were enrolled in the final analysis (supplementary figure). Data were obtained from the National Health and Nutrition Examination Survey (NHANES, years from 2015 to 2018), which is designed to assess the health and nutritional status of the noninstitutionalized, civilian, US population. The NHANES interview includes demographic, socioeconomic, and health‐related questions. The examination component consists of medical, dental, and physiological measurements as well as laboratory tests administered by highly trained medical personnel. The protocols for the conduct of NHANES were approved by the National Center for Health Statistics institutional review board (NCHS IRB/ERB), and informed consent was obtained from all participants. The Ethics Review Board for the National Center for Health Statistics (NCHS ERB) approved the NHANES. The data and study materials of the NHANES project are open to everyone and freely available at http://www.cdc.gov/nchs/nhanes.

### 2.2 Assessment of depressive symptoms

Depressive symptoms were measured using the Patient Health Questionnaire‐9 (PHQ‐9) (Gilbody et al., 2007). Participants were asked to choose one of four responses about the frequency of depressive symptoms during the previous 2 weeks. The PHQ‐9 has nine items, each item on the form was scored on a scale of 0 to 3, and total scores ranged from 0 to 27, with a higher score indicating higher depressive symptoms. PHQ‐9 scores of 10 or higher have been validated as a cut‐off to indicate depression (Manea et al., 2012).

### 2.3 Assessment of MetS

MetS was diagnosed if at least three of the following five items of the National Cholesterol Education Program, Adult Treatment Panel III (NCEP ATP III) criteria were met (Grundy et al., 2005):

1. Fasting triglyceride level ≥1.7 mmol/L or treated with drugs,
2. HDL‐C levels < 1.1 mmol/L for women and < 0.9 mmol/L for men or treated with drugs,
3. Fasting blood glucose ≥5.6 mmol/L, treated with drugs or previously diagnosed with type 2 diabetes mellitus.
4. Increased blood pressure: systolic blood pressure (SBP) ≥130 mmHg, diastolic blood pressure (DBP) ≥ 85 mmHg, treated with antihypertensive drugs, or previously diagnosed with hypertension; and
5. Central obesity: waist circumference ≥90 cm for men and ≥80 cm for women.

### 2.4 Assessment of covariates

Based on the existing literature, variables that might potentially confound the relationship between LHR and depression were assessed in the analysis: sex, age, race/ethnicity, education level, smoking status, drinking status, marital status, family poverty ratios (as an indicator of household income level), body mass index (BMI), diabetes mellitus, hypertension, cancer, antidepressant, antihypertension treatment, hypoglycemic treatment, lipid‐lowering therapy, and sedentary behavior. race/ethnicity was classified as non‐Hispanic white, non‐Hispanic black, Mexican American, or other. Education was categorized as less than high school or at least high school education. Smoking status was assessed by responses to: “Do you now smoke cigarettes?” participants who answered “some days” or “every day” were classified as current smokers. Drinking status was established by responses to: “During the past 12 months, about how often did you drink any type of alcoholic beverage?,” participants who answered “at least 3 times” were classified as current drinking. Marital status was classified into two groups: live with someone (married or living with a partner) and live alone (never married, separated, divorced, and widowed). Family poverty ratios were classified into <1 (lower income), 1–4 (middle income), and ≥4 (higher income). BMI was calculated as weight/height squared (kg/m^2^) and divided into three groups: normal < 25, overweight 25–30, or obese ≥30. Doctor‐diagnosed medical comorbidities, including diabetes mellitus and hypertension, were identified by asking participants: “Have ever been told by a doctor or health professional that you have _____”. Sedentary behavior was assessed by responses to “How much time do you usually spend sitting on a typical day?”. LHR was defined as the ratio of lymphocyte counts to HDL‐C.

### 2.5 Statistical analysis

Population characteristics are presented as means ± SD for continuous variables and percentages for categorical variables. Comparisons in the distribution of characteristics according to depressive symptoms were performed by the student's *t*‐test or the Mann–Whitney *U* test for continuous variables and the χ2 test for categorical variables.

Multivariable logistic regression models (odds ratio (OR) and 95% confidence intervals (95% CIs)) were used to evaluate the association between LHR and depression with adjustment for age, sex, and race in model 1 and sex, age, race, education, smoking status, drinking status, marital status, family poverty ratios, BMI, diabetes mellitus, hypertension, cancer, antidepressant, antihypertension treatment, hypoglycemic treatment,  lipid‐lowering therapy, and sedentary behavior in model 2. The smooth curve fittings and generalized additive models were used to assess the relationship between LHR and depression after adjusting for the potential confounders. Observations of LHR exceeding 3 standard deviations from the mean were classified as outliers, and a sensitivity analysis was conducted to evaluate the association between LHR and depression using multivariable logistic regression models on the data after excluding these outliers.

Furthermore, possible modifications of the relationship of depression (high vs. low) with LHR were explored for the following variables: age (< 60 vs. ≥60), sex, BMI (< 25 vs. 25–30 vs. ≥30), smoking status (no vs. yes), drinking status (no vs. yes), marital status (live with someone vs. live alone), diabetes (no vs. yes), hypertension (no vs. yes), cancer (no vs. yes), MetS (no vs. yes), hsCRP (<3 mg/L vs. ≥3 mg/L) by stratified analyses and interaction testing.

Mobile Examination Center sample weights and the appropriate home‐examined sample design variables (strata, primary sampling unit) were used in the analysis to account for the complex survey design (including oversampling) and survey nonresponse and were poststratified to obtain nationally representative estimates of the U.S. civilian noninstitutionalized population using the R package survey. All data analyses were performed by GraphPad Prism version 9 (GraphPad Software Inc.), EmpowerStats (http://www.empowerstats.net/cn/index.php), and R software, version 4.2.2 (http://www.r‐project.org). A two‐tailed *p*‐value of less than .05 was considered statistically significant.

## 3 RESULTS

### 3.1 Baseline characteristics of the participants

A total of 4,216 participants were included in the final analysis (Supplementary Figure 1). The baseline characteristics of the study population by depressive symptoms categories (undepressed or depressed) are shown in Table 1. The mean age of this population was 51.5 years, 41.9% were males, 12.4% were classified as having high depressive symptoms, 25.7% were overweight, 47.2% were obese and 68% were Non‐Hispanic White. Participants with high depressive symptoms were more likely to be female and live alone; were more likely to be currently drinking, currently smoking, overweight, and obese; had a lower level of education and economic; had a lower percentage of hypertension, lipid‐lowering therapy and hypoglycemic treatment; had a higher percentage of diabetes overweight, cancer, MetS, and antidepressant; had lower albumin, creatinine, HDL cholesterol, and PHQ‐9; and had higher lymphocyte count, hsCRP, glucose, triglyceride, and lymphocyte to HDL‐C ratio (LHR).

**TABLE 1:** Baseline characteristics of the study participants.

		Depressive symptoms		
Variables	Overall	Low, <10	high, ≥10	*p*‐value
*N*	4216	3695	521	
Age, year	51.5 ± 20.5	55.9 ± 17.2	55.1 ± 15.8	.32
Male	41.9	42.1	22.8	<.001
Race				<.001
Mexican American	7	6.1	7.1	
Non‐Hispanic White	68	68.9	77.5	
Non‐Hispanic Black	11.1	11.4	8.1	
Other	13.9	13.6	7.3	
Education				.01
< 12 years	15.7	13.4	17.7	
≥ 12 years	84.3	86.6	82.3	
Marriage status				<.001
Live alone	40.3	38.1	50	
Live with someone	59.7	61.9	50	
Family poverty ratio				<.001
<1	15.4	13.1	30.3	
1–4	48	45.8	53.8	
≥ 4	36.6	41.2	15.9	
Current alcohol use	26.9	25.4	41.4	<.001
Current smoking	37.6	32.8	60.2	<.001
Diabetes	23	24.3	34.3	<.001
Hypertension	83.6	84.7	75.1	<.001
Cancer	15.7	13.9	16.9	.01
MetS	26.2	25.3	32.6	<.001
Antidepressant	0.78	0.57	2.65	<.001
Antihypertension treatment	73.8	74	70.8	.05
Hypoglycemic treatment	66.3	65.8	59.6	.01
Lipid‐lowering therapy	84.1	84.7	78	.01
BMI categories				<.001
<25	27.1	23.4	13.4	
25–30	25.7	25.7	33.2	
≥ 30	47.2	50.9	53.4	
Sedentary behavior, in minutes per day	527.4 ± 1073.1	525.2 ± 1135.2	540.5 ± 938.9	.77
Lymphocyte count, 109/L	2.2 ± 0.7	2.1 ± 0.7	2.4 ± 0.8	<.001
Hemoglobin, g/L	13.9 ± 1.5	13.9 ± 1.6	13.9 ± 1.3	.95
Albumin, g/L	42.8 ± 3.6	42.9 ± 3.5	41.7 ± 3.5	<.001
Creatinine, umol/L	79.0 ± 43.5	80.9 ± 47.1	76.2 ± 28.4	.02
hsCRP, mg/L	5.1 ± 10.2	4.5 ± 8.1	8.9 ± 18.1	<.001
Glucose, mmol/L	6.0 ± 2.3	6.0 ± 2.3	6.4 ± 2.5	<.001
HDL cholesterol, mmol/L	1.4 ± 0.4	1.4 ± 0.5	1.3 ± 0.4	.001
Triglyceride, mmol/L	1.8 ± 1.3	1.8 ± 1.3	2.0 ± 0.9	.002
HbA1c, %	5.9 ± 1.2	6.0 ± 1.2	6.1 ± 1.1	.2
Uric acid, mg/dl	5.5 ± 1.6	5.5 ± 1.6	5.7 ± 1.5	.04
PHQ‐9	4.5 ± 4.9	2.8 ± 2.7	14.5 ± 3.6	<.001
Lymphocyte to HDL‐C ratio	1.7 ± 0.9	1.7 ± 0.9	1.9 ± 0.8	<.001

*Note*: Data are expressed as mean ± SD and percentages.

Abbreviations: BMI, body mass index; HDL, HIgh‐density lipoprotein; hsCRP, high‐sensitive C‐reactive protein; MetS, metabolic syndrome.; PHQ‐9: Patient Health Questionnaire‐9.

### 3.2 Association between the prevalence of depression and LHR

**FIGURE 1:**
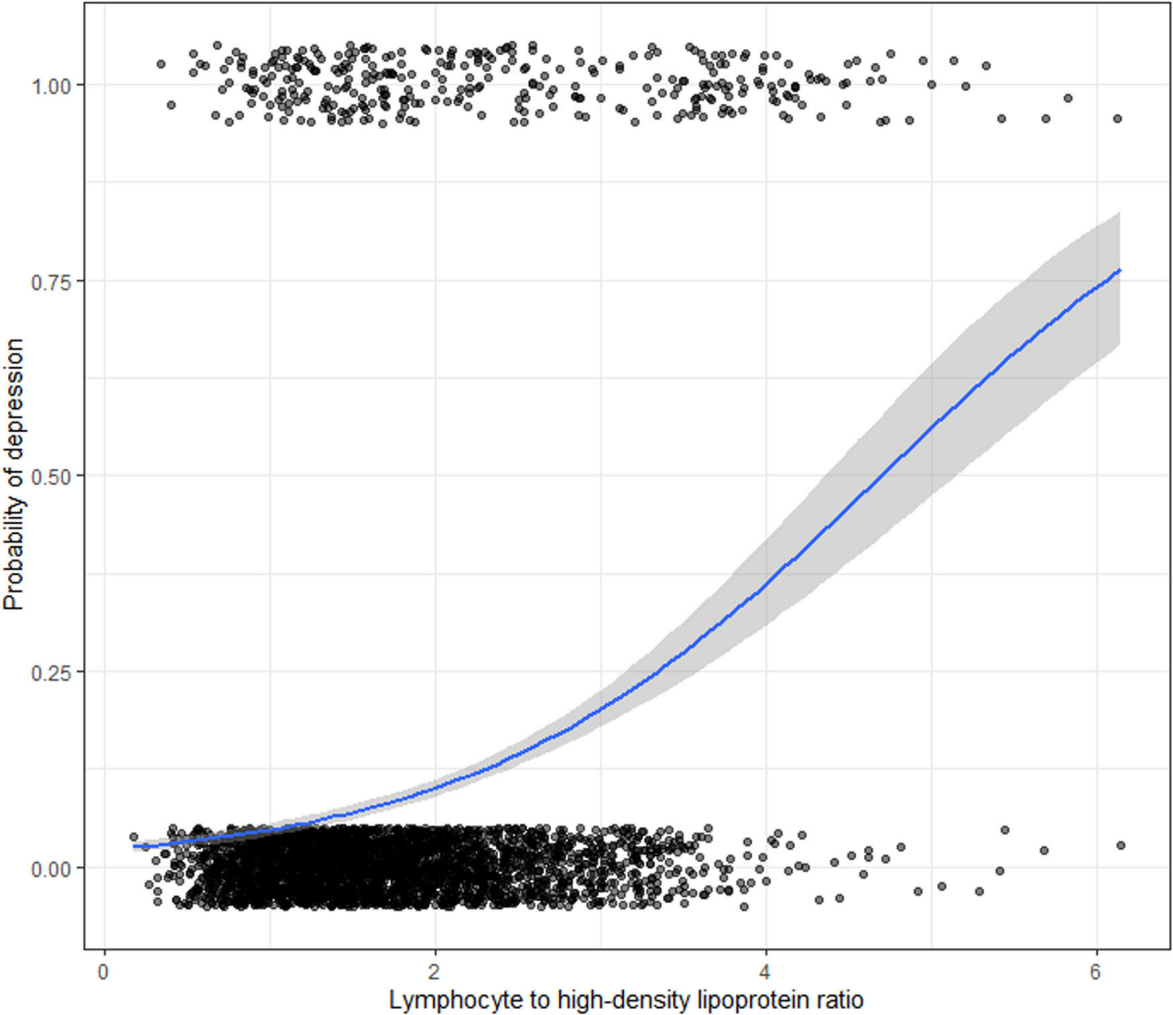
The relationship between LHR and depression**^.^** Adjusted for sex, age, race, education, smoking status, drinking status, marital status, family poverty ratios, BMI, diabetes mellitus, hypertension, cancer, antidepressant, antihypertension treatment, hypoglycemic treatment,  lipid‐lowering therapy and sedentary behavior. LHR: lymphocyte to high‐density lipoprotein ratio; BMI: body mass index.

The association between depression and LHR is plotted in Figure 1. As shown in Table 2, after adjustment for major demographic (model 1), there was a significant positive association between depression and LHR (per standard deviation (SD) increment; adjusted OR, 1.43; 95% CI [1.30, 1.57]). Similar results were found in the fully adjusted model (model 2). In addition, we performed a sensitivity analysis to assess the relationship between LHR and depression using multivariable logistic regression models on the outlier‐free data and obtained results consistent with those from the entire dataset (adjusted OR, 1.40; 95% CI [1.21, 1.62], Supplementary Table 4 and Supplementary Figure 2).

**TABLE 2:** The associations between LHR and depression.

Exposure	Crude	*p*‐value	Model 1	*p*‐value	Model 2	*p*‐value
	OR (95% CI)		OR (95% CI)		OR (95% CI)	
LHR, per SD increment	1.35 [1.24, 1.48]	<.001	1.43 [1.30, 1.57]	<.001	1.31 [1.14, 1.50]	<.001

*Note*: Model 1 was adjusted for age, sex, and race. Model 2 was adjusted for sex, age, race, education, smoking status, drinking status, marital status, family poverty ratios, BMI, diabetes mellitus, hypertension, cancer, antidepressant, antihypertension treatment, hypoglycemic treatment,  lipid‐lowering therapy and sedentary behavior.

Abbreviations: LHR: lymphocyte to high‐density lipoprotein ratio; SD, standard deviation.

The associations of specific depressive symptoms and LHR are presented in Supplemental Table 2. Only seven symptoms (felt everything was an effort: adjusted OR, 1.12; 95% CI [1.01, 1.24]; felt depressed: adjusted OR, 1.34; 95% CI [1.21, 1.50]; poor appetite or overeating: adjusted OR, 1.22; 95% CI [1.10, 1.36]; feeling bad about yourself: adjusted OR, 1.19; 95% CI [1.05, 1.34]; had trouble concentrating: adjusted OR, 1.18; 95% CI [1.04, 1.33]; moving or speaking slowly or too fast: adjusted OR, 1.20; 95% CI [1.06, 1.36]) and thought you would be better off dead: adjusted OR, 1.65; 95% CI [1.35, 2.02]) were significantly associated with LHR.

### 3.3 Stratified analyses

**FIGURE 2:**
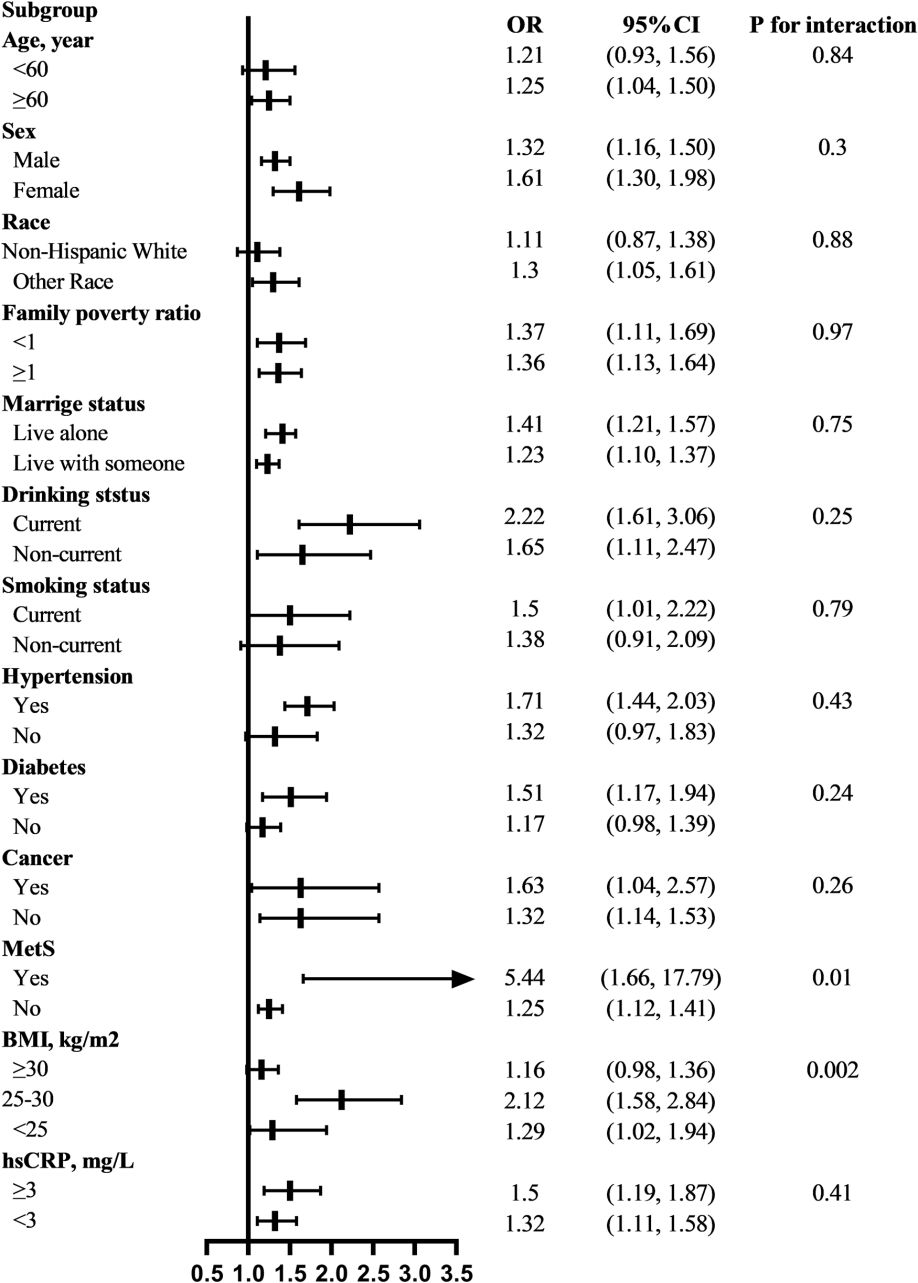
Stratified analyses for the association between LHR and depression (high vs. low). Adjusted for sex, age, race, education, smoking status, drinking status, marital status, family poverty ratios, BMI, diabetes mellitus, hypertension, cancer, antidepressant, antihypertension treatment, hypoglycemic treatment,  lipid‐lowering therapy and sedentary behavior. BMI: body mass index; CI, confidence interval; hsCRP, high‐sensitivity C‐reactive protein; LHR: lymphocyte to high‐density lipoprotein ratio; OR, odds ratio.

Stratified analyses were further performed to evaluate the association between LHR and depression in various subgroups (Figure 2). Interactions between LHR with MetS (*p* for interaction: .02) and BMI (*p* for interaction: .01) on the risk of depression were found in this study. None of these variables, including age, sex, race, family poverty ratio, marriage status, drinking status, smoking status, hypertension, diabetes, cancers, and high‐sensitivity CRP, significantly modified the associations between the LHR, and the risk of depression (*p* for interaction >.05 for all comparisons).

## 4 DISCUSSION

In this national representative cross‐sectional study, we first demonstrated a higher

level of LHR was associated with higher odds of having depression in US adults. Interactions between LHR with MetS and BMI on the risk of depression were found in stratified analysis.

The possible explanations for the relationship between LHR and depression remain to be further elucidated; we speculate that there are several potential mechanisms that may explain the results of this study. First, the association between LHR and depression may be mediated by insulin resistance (IR). The relationship between IR and depression has been reported by previous studies in general populations (Kan et al., 2013; Lee et al., 2017; Pan et al., 2008; Pearson et al., 2010). A cross‐sectional study with a total of 165,443 participants in South Korea showed that a significant positive relation was observed between IR and depression in general populations. With the increment of IR, the odds of having depression in young adults and nondiabetic participants increase by 4% and 17%, respectively (Lee et al., 2017). Pan et al. (2008) conducted a cross‐sectional study in China, with a total of 3285 participants aged 50−70 years, which showed that participants with depressive symptoms had a higher risk of having IR (OR = 1.54, 95% CI [1.17, 2.04]). Moreover, a cross‐sectional study conducted by Pearson et al. (2010) in Australia, with a total of 1732 participants aged from 26 to 36 years, showed that a higher risk of depression was associated with IR. With the increment of IR, the risk of depression in men and women increases by 13.2% and 6.1%, respectively. Furthermore, IR is also the most important pathway in the pathophysiology of MetS (Eckel et al., 2005). An interaction between LHR with MetS on the risk of depression was found in this study (Figure 2). Participants with MetS were more likely to have depression compared to those without MetS (*p* for interaction: .02). LHR has been reported to be a potential marker of inflammation (H. Chen et al., 2019; T. Chen et al., 2020) that can predict the occurrence and severity of MetS (Yu et al., 2021). Cytokines and proteolytic enzymes induced by chronic inflammation, insulin, and insulin growth factors, I and II may explain the association between LHR and MetS (Lao et al., 2008; Pasini et al., 2010; Tamariz et al., 2008). At the same time, previous studies have found that lymphocytes proliferate and participate in the pathogenesis of IR (Piatkiewicz et al., 2010; Stentz and Kitabchi, 2007). HDL‐C has anti‐inflammatory and antioxidant properties that prevent the migration of macrophages in atherosclerosis and promote the export of oxidized low‐density lipoprotein cholesterol (LDL‐C) (Akboga et al., 2016; Wu et al., 2013) and is a protective factor in MetS (Rohatgi, 2015; Wu et al., 2013). Moreover, lower serum HDL‐C levels were associated with higher odds of having depression, and the immune/inflammatory response may contribute to lower serum HDL‐C in depressive populations (Maes et al., 1997). Furthermore, the LHR was significantly associated with psychiatric diseases according to a previous study (Wei et al., 2022). Second, inflammatory and oxidative stress might be another reason contributing to the relationship between LHR and depression. A growing body of evidence reported that proinflammatory cytokines like interleukin (IL)−6 and TNF and acute phase proteins like CRP are increased in depressive patients in the blood compared to healthy controls (Dowlati et al., 2010; Howren et al., 2009; Köhler et al., 2017; Liu et al., 2012; Maes et al., 2009; Miller et al., 2009). A meta‐analysis of 82 studies involving 3212 depressive participants and 2798 healthy controls suggested increased levels of IL‐6, tumor necrosis factor (TNF), IL‐10, IL‐13, IL‐18, IL‐12, and soluble TNF receptor 2 in depressive patients (Köhler et al., 2017). Cytokine inhibitor monotherapies can ameliorate depression (Kappelmann et al., 2018; Köhler et al., 2019) and antidepressant treatment also significantly decreases peripheral levels of IL‐6, TNF, and IL‐10 (Köhler et al., 2018). LHR could act as an index of inflammatory status according to previous studies (H. Chen et al., 2019; T. Chen et al., 2020; Yu et al., 2021). Both inflammation and oxidative stress can cause vascular endothelial damage, which may result in the occurrence and progression of a variety of diseases, such as dementia and vascular depression (Luca and Luca, 2019). Consistently, compared with nondepressive participants, levels of CRP and lymphocyte counts are higher in depressive participants in our study. Moreover, stratified analysis in this study suggests that hsCRP did not significantly modify the associations between the LHR and the risk of depression (*p* for interaction >.05), and LHR was associated with depression in different categories of hsCRP (Figure 2). CRP is easily available and is most commonly used in clinical practice. However, the magnitude of the association between CRP and depression is still unclear due to the significant variability in the methods of controlling potential confounders and quality measures (Horna et al., 2018). More comprehensive studies are needed in the future to quantify the relationship between CRP and depression. What's more, an interaction between LHR and BMI on the risk of depression was found in this study (Figure 2). Participants with BMI ranges from 25 to 30 had the highest OR, and the 95% CI between the lowest category of BMI (< 25 kg/m^2^) and the middle (25–30 kg/m^2^) did not overlap, which might be explained by central obesity. Because participants with a BMI range from 25 to 30 had the highest prevalence of central obesity in this study (Supplementary Table 3), and central obesity is correlated with inflammation as a previous study has reported (Haffner, 2007).

Third, immune dysfunction may also play a key role in the association of LHR with depression. It is well known to us that HDL‐C is the plasma lipoprotein responsible for reverse cholesterol transport, which is a protective factor in the context of atherosclerosis. Moreover, HDL‐C also plays an important role in the modulation of the immune response (Fernandes das Neves et al., 2021). Previous studies reported that HDL‐C can increase the proliferation of stimulated T cells in healthy young individuals (Larbi et al., 2014). and HDL‐C was associated with immune/inflammatory markers, such as the (CD4+/CD8+) T‐cell ratio (Maes et al., 1997). At the same time, depression has been reported to be associated with the activation of the immune system, including abnormality in antibody titers immune cell numbers, and inflammatory markers (Gibney and Drexhage 2013, Müller, 2014). There has been an observation that the number of leukocytes, neutrophils, and monocytes, the ratio of CD4/ CD8 T‐cells, and the activation of monocytes/ macrophages were seen to increase in depressive patients (Beurel et al., 2020). Further studies are needed to explore the details of the mechanism explaining the association between LHR and depression.

A good biomarker should be cost‐effective and easily reproducible with good sensitivity and specificity and can be used to screen the condition early and track its severity. Although serum cytokines are important markers of depression for their roles in its pathogenesis, diagnosis, and management (Harsanyi et al., 2023), they are expensive to measure and hard to monitor routinely in clinical practice. LHR is an inexpensive and easily available marker in clinical practice. As a combined indicator, it might be more reliable in reflecting the inflammation level and the body's immune than a single parameter because of its complex interactions between lymphocytes and HDL‐C. To our knowledge, although the ability to diagnose depression between LHR and other biomarkers has not been compared in previous studies, LHR was superior to hsCRP and lymphocyte counts in diagnosing the presence of MetS (H. Chen et al., 2019). A longitudinal study with 1194 participants in China reported that LHR was superior to the platelet‐to‐lymphocyte ratio after a 4.66‐year follow‐up (Yu et al., 2021).

The strengths of this study included the ability to examine the relationship between LHR and depression in a large and nationally representative population. The finding that LHR was significantly associated with depression provides further evidence for the bidirectional relationship between depression and inflammation/MetS, which suggests LHR might be a novel hallmark of depression. LHR is a more easily accessible and less expensive inflammation marker than others such as cytokines. Several limitations should be considered in our study. First, depressive symptoms were only assessed using PHQ‐9, which is not the gold standard for the clinical diagnosis of depression. However, PHQ‐9 has been proven to be an efficient and valid tool for screening major depression (Levis et al., 2019). Second, although most of the potential confounders had been considered, residual confounding, such as baseline active infection, immunological diseases, percent body fat, and use of birth control in females, may not be completely considered. However, the comprehensive data on the risk factors minimized potential confounding. Third, this is a cross‐sectional study, we are unable to establish causality between LHR and depression. Fourth, some of the participants were excluded from this analysis due to incomplete depression measurement and blood data. And we did a comparison of baseline characteristics between participants excluded and those included. Results showed characteristics of the participants excluded did not differ from those included except for “race, education, and family poverty ratio” (Supplementary Table 1). We admitted there might have been a selection bias; however, we used different models to assess the association between LHR and depression with the adjustment of multiple variables, and results were consistent in different models with different categories of LHR. Moreover, similar results were obtained in stratified analysis. Overall, further studies are needed to verify our results in the future.

## 5 CONCLUSIONS

In summary, our study demonstrated that LHR was significantly associated with depression in U.S. adults, and it was strengthened in participants with MetS and BMI ranging from 25 to 30 kg/m^2^. This finding suggests LHR might be an independent indicator of depression development. If further confirmed, it would be a simple and effective way for depression screening and to improve the treatment of depression in the early stage.

## AUTHOR CONTRIBUTIONS

**Junzhi Chen**: Conceptualization; data curation; formal analysis; methodology; supervision; validation; visualization; writing—original draft; writing—review and editing. **Yan Huang**: Data curation; formal analysis; methodology; validation; visualization; writing—review and editing. **Xiaolin Li**: Data curation; formal analysis; methodology; validation; visualization.

## CONFLICT OF INTEREST STATEMENT

No disclosure was reported.

## FUNDING INFORMATION

This research did not receive any specific grant from funding agencies in the public, commercial, or not‐for‐profit sectors.

### PEER REVIEW

The peer review history for this article is available at https://publons.com/publon/10.1002/brb3.3467.

## Supporting information

Supplementary Figure 1 Flow diagram indicating a derivation of the final analyzed study sample.
Supplementary Figure 2 The relationship between LHR and depression*
* Adjusted for sex, age, race, education, smoking status, drinking status, marital status, family poverty ratios, BMI, diabetes mellitus, hypertension, cancer, antidepressant, antihypertension treatment, hypoglycemic treatment,  lipid‐lowering therapy , and sedentary behavior. Abbreviations: LHR: lymphocyte to high‐density lipoproten ratio; BMI: body mass index.
Supplementary table 1 Characteristics of the included and excluded participants.
Supplementary table 2 The association between specific LHR and depressive symptoms *
* Adjusted for sex, age, race, education, smoking status, drinking status, marital status, family poverty ratios, BMI, diabetes mellitus, hypertension, cancer, antidepressant, antihypertension treatment, hypoglycemic treatment,  lipid‐lowering therapy and sedentary behavior. Abbreviations: LHR: lymphocyte to high‐density lipoprotein ratio; BMI: body mass index.
Supplementary table 3 Characteristic of central obesity in baseline participants stratified by BMI and depression.
Supplementary table 4 The associations between LHR and depression.

## Data Availability

The data that support the findings of this study are openly available in the National Health and Nutrition Examination Survey at http://www.cdc.gov/nchs/nhanes.
