# Supplementary material for: The association between lymphocyte to high‐density lipoprotein ratio and depression: Data from NHANES 2015–2018
Source: Brain Behav. 2024 Mar 11;14(3):e3467. doi: 10.1002/brb3.3467 (PMC10928332; doi:10.1002/brb3.3467)
Supplement: Supplementary file 1 — Supplementary Figure 1 Flow diagram indicating a derivation of the final analyzed study sample. Supplementary Figure 2 The relationship between LHR and depression* * Adjusted for sex, age, race, education, smoking status, drinking status, marital status, family poverty ratios, BMI, diabetes mellitus, hypertension, cancer, antidepressant, antihypertension treatment, hypoglycemic treatment, lipid‐lowering therapy , and sedentary behavior. Abbreviations: LHR: lymphocyte to high‐density lipoproten ratio; BMI: body mass index. Supplementary table 1 Characteristics of the included and excluded participants. Supplementary table 2 The association between specific LHR and depressive symptoms * * Adjusted for sex, age, race, education, smoking status, drinking status, marital status, family poverty ratios, BMI, diabetes mellitus, hypertension, cancer, antidepressant, antihypertension treatment, hypoglycemic treatment, lipid‐lowering therapy and sedentary behavior. Abbreviations: LHR: lymphocyte to high‐density lipoprotein ratio; BMI: body mass index. Supplementary table 3 Characteristic of central obesity in baseline participants stratified by BMI and depression. Supplementary table 4 The associations between LHR and depression. [file BRB3-14-e3467-s001.docx]

**
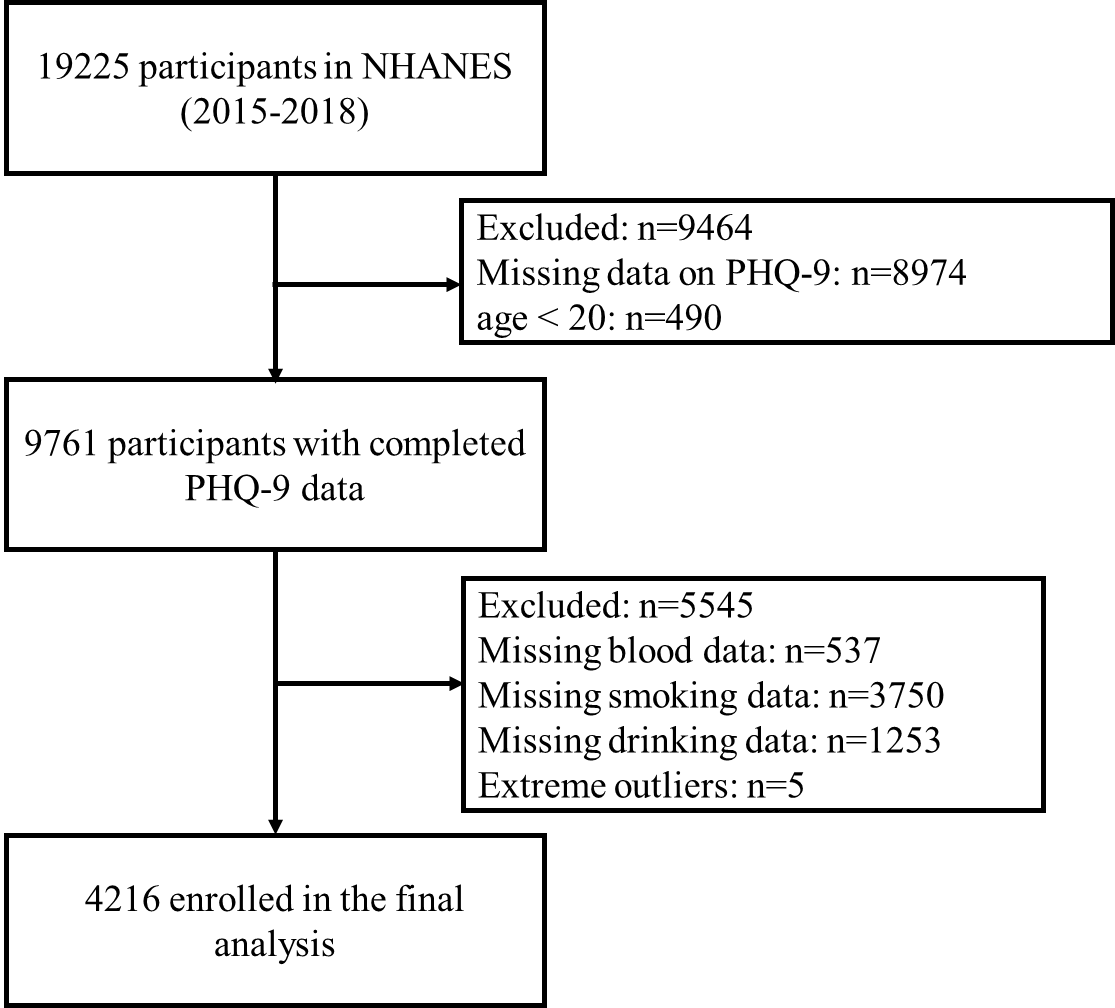
**

**Supplementary figure 1.** **Flow diagram indicating derivation of final analyzed study sample**

**Supplementary figure 2 The relationship between LHR and depression^*^**


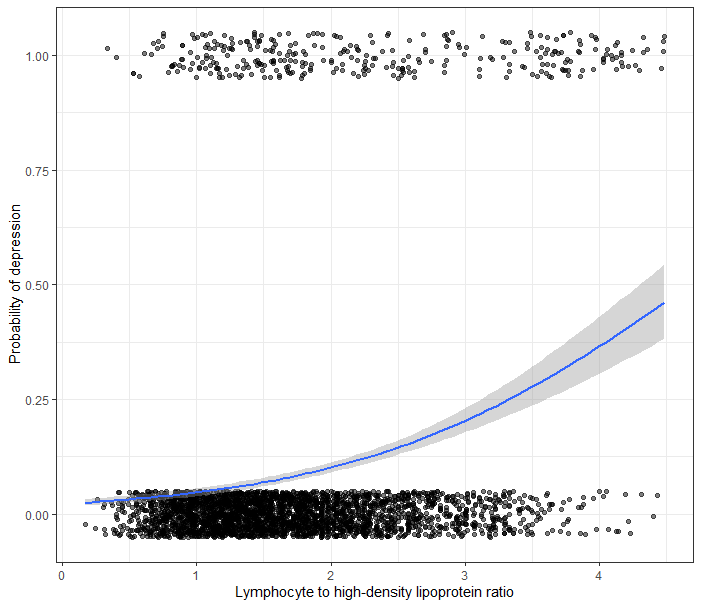


^*^ Adjusted for sex, age, race, education, smoking status, drinking status, marital status, family poverty ratios, BMI, diabetes mellitus, hypertension, cancer, antidepressant, antihypertension treatment, hypoglycemic treatment,  lipid‐lowering therapy and sedentary behavior. **Abbreviations:** LHR: lymphocyte to high-density lipoprotein ratio; BMI: body mass index.

**Supplementary table 1. Characteristics of the included and excluded participants**

| Variables | Excluded | Included |
| --- | --- | --- |
| N | 15004 | 4216 |
| Age, year | 51.1 ± 22.8 | 51.5 ± 20.5 |
| Male | 44 | 41.9 |
| Race |  |  |
| Mexican American | 10.6 | 7 |
| Non-Hispanic White | 17.4 | 68 |
| Non-Hispanic Black | 59.9 | 11.1 |
| Other | 12.1 | 13.9 |
| Education |  |  |
| < 12 years | 10 | 15.7 |
| ≥ 12 years | 90 | 84.3 |
| Marrige status |  |  |
| Live alone | 37.1 | 40.3 |
| Live with someone | 62.9 | 59.7 |
| Family poverty ratio |  |  |
| <1 | 24.3 | 15.4 |
| 1-4 | 53.9 | 48 |
| >=4 | 21.9 | 36.6 |
| Current alcohol use | 28.9 | 26.9 |
| Current smoking | 37.3 | 37.6 |
| Diabetes | 23.6 | 23 |
| Hypertension | 84.2 | 83.6 |
| BMI categories |  | 27.1 |
| Normal weight | 28.9 | 25.7 |
| Overweight | 71.1 | 47.2 |
| Sedentary Behavior, in minutes per day | 490.9 ± 953.9 | 527.4 ± 1073.1 |
| Lymphocyte count, 109/L | 2.4 ± 1.5 | 2.2 ± 0.7 |
| Hemoglobin, g/L | 14.0 ± 1.5 | 13.9 ± 1.5 |
| Albumin, g/L | 42.4 ± 3.7 | 42.8 ± 3.6 |
| Creatinine, umol/L | 83.5 ± 57.1 | 79.0 ± 43.5 |
| hsCRP, mg/L | 5.5 ± 12.9 | 5.1 ± 10.2 |
| Glucose, mmol/L | 6.3 ± 2.9 | 6.0 ± 2.3 |
| HDL cholesterol, mmol/L | 1.4 ± 0.4 | 1.4 ± 0.4 |
| Triglyceride, mmol/L | 1.6 ± 1.2 | 1.8 ± 1.3 |
| HbA1c, % | 6.2 ± 1.4 | 5.9 ± 1.2 |
| Uric acid, mg/dl | 5.4 ± 1.4 | 5.5 ± 1.6 |
| Lymphocyte to HDL-C ratio | 1.9 ± 1.5 | 1.7 ± 0.9 |

**Abbreviations:** BMI: body mass index; hsCRP: high-sensitive C-reactive protein; PHQ-9: patient health questionnaire-9; HDL: High-density lipoprotein.

**Supplementary table 2. The association between specific LHR and depressive symptoms** ^*^

| **Depressive symptoms** | **Crude** | **Model 1** | **Model 2** |
| --- | --- | --- | --- |
|  |  | **OR (95%CI)** | **OR (95%CI)** |
| Felt everything was an effort | 1.26 (1.18, 1.35) | 1.29 (1.21, 1.39) | 1.12 (1.01, 1.24) |
| Felt depressed | 1.25 (1.17, 1.34) | 1.30 (1.21, 1.40) | 1.34 (1.21, 1.50) |
| Sleep was restless | 1.04 (0.97, 1.10) | 1.05 (0.99, 1.13) | 0.84 (0.76, 1.23) |
| Feeling tired or having little energy | 1.11 (1.04, 1.19) | 1.18 (1.10, 1.26) | 1.04 (0.94, 1.15) |
| Poor appetite or overeating | 1.32 (1.24, 1.42) | 1.36 (1.26, 1.46) | 1.22 (1.10, 1.36) |
| Feeling bad about yourself | 1.23 (1.14, 1.33) | 1.23 (1.13, 1.33) | 1.19 (1.05, 1.34) |
| Had trouble concentrating | 1.14 (1.05, 1.23) | 1.15 (1.06, 1.25) | 1.18 (1.04, 1.33) |
| Moving or speaking slowly or too fast | 1.18 (1.08, 1.28) | 1.23 (1.13, 1.34) | 1.20 (1.06, 1.36) |
| Thought you would be better off dead | 1.28 (1.12, 1.46) | 1.27 (1.11, 1.46) | 1.65 (1.35, 2.02) |

^*^ Adjusted for sex, age, race, education, smoking status, drinking status, marital status, family poverty ratios, BMI, diabetes mellitus, hypertension, cancer, antidepressant, antihypertension treatment, hypoglycemic treatment,  lipid‐lowering therapy and sedentary behavior. **Abbreviations**: LHR: lymphocyte to high-density lipoprotein ratio; BMI: body mass index.

**Supplementary table 3. Characteristic of central obesity in baseline participants stratified by BMI and depression.**

| **Variables** | | **Undepressed, <10 (N = 3695)** | **Depressed, >= 10 (N = 521)** |
| --- | --- | --- | --- |
| Central obesity | BMI < 25 kg/m(2) | 48.7% | 45.8% |
|  | BMI 25-30 kg/m(2) | 89.4% | 98.1% |
|  | BMI >= 30 kg/m(2) | 79.3% | 83.3% |

**Abbreviations**: BMI: body mass index.

**Supplementary table 4. The associations between LHR and depression.**

| **Exposure** | **Crude** |  | **Model 1** | **P value** | **Model 2** | **P value** |
| --- | --- | --- | --- | --- | --- | --- |
|  |  |  | **OR (95%CI)** |  | **OR (95%CI)** |  |
| **LHR, per SD increment** | 1.47 (1.34, 1.62) | <0.001 | 1.56 (1.41, 1.72) | <0.001 | 1.40 (1.21, 1.62) | <0.001 |

Adjusted for sex, age, race, education, smoking status, drinking status, marital status, family poverty ratios, BMI, diabetes mellitus, hypertension, cancer, antidepressant, antihypertension treatment, hypoglycemic treatment,  lipid‐lowering therapy and sedentary behavior.
